# Resolution of disseminated fusariosis in a child with acute leukemia treated with combined antifungal therapy: a case report

**DOI:** 10.1186/1471-2334-7-40

**Published:** 2007-05-10

**Authors:** José-Manuel Vagace, Cesar Sanz-Rodriguez, Maria-Soledad Casado, Nieves Alonso, Manuel Garcia-Dominguez, Francisco Garcia de la Llana, Luis Zarallo, Miguel Fajardo, Roberto Bajo

**Affiliations:** 1 Department of Hematology, Complejo Hospitalario Infanta Cristina, Badajoz, Spain; 2 Department of Clinical Research, Merck Sharp & Dohme of Spain, Madrid, Spain; 3 Department of Internal Medicine, Complejo Hospitalario Infanta Cristina, Badajoz, Spain; 4 Department of Pediatrics, Complejo Hospitalario Infanta Cristina, Badajoz, Spain; 5 Department of Microbiology, Complejo Hospitalario Infanta Cristina, Badajoz, Spain

## Abstract

**Background:**

*Fusarium *spp. is being isolated with increasing frequency as a pathogen in oncohematologic patients. Caspofungin and amphotericin B have been reported to have synergistic activity against *Fusarium *spp.

**Case presentation:**

We herein report a case of disseminated fusariosis diagnosed by chest CT scan and positive blood cultures to *Fusarium *spp. Because the patient's clinical condition deteriorated, CRP levels increased, and blood cultures continued to yield *Fusarium *spp. despite liposomal amphotericin B monotherapy up to 5 mg/kg daily, treatment with caspofungin was added. Within 2 weeks of onset of combined antifungal therapy, the chest CT scan demonstrated a progressive resolution of the pulmonary lesions. Upon discontinuation of intravenous antifungals, the patient received suppressive therapy with oral voriconazole. Three months later, a chest CT scan showed no abnormalities. Twenty-five months after discontinuation of all antifungal therapy, the patient remains in complete remission of her neoplastic disease with no signs of clinical activity of the *Fusarium *infection.

**Conclusion:**

This is the first description of successful treatment of disseminated fusariosis in a pediatric patient with acute lymphoblastic leukemia with caspofungin and amphotericin B followed by oral suppressive therapy with voriconazole.

## Background

*Fusarium *spp. is a saprophytic mould that is ubiquitous in the soil. Invasive fusariosis may follow the inhalation of airborne conidia or the inoculation of conidia through a skin breach associated with indwelling catheters, wounds, burns, or onychomycosis. Deep-seated and disseminated infections caused by *Fusarium *spp. are being diagnosed with increasing frequency in patients with hematological malignancies [1]. The *Fusarium *species most frequently involved in human infections are *Fusarium solani*, *F. oxysporum*, *F. verticilloides*, and *F. moniliforme *[1]. Of note, *Fusarium *species are often confused with *Aspergillus *spp. as both pathogens have similar histopathologic appearance with septate, dichotomously branching hyphae. Yet, the prognosis of invasive fusariosis is much poorer than for invasive aspergillosis, with very low response rates and most patients with disseminated *Fusarium *infection dying [1]. We describe here the first case of a favorable response of disseminated fusariosis to combination caspofungin and liposomal amphotericin B followed by oral suppressive therapy with voriconazole in a pediatric patient with acute lymphoblastic leukaemia (ALL).

## Case presentation

A 11-year-old, 50-kg female with a diagnosis of CD10+ ALL was admitted to the hospital with fever and intermittent chest pain. The patient was in complete remission (CR) following SHOP-99 chemotherapy. At the time of hospital admission, she was receiving G-CSF for severe granulocytopenia post-intensification with cytarabine, and had also received broad-spectrum antibiotics and fluconazole prophylaxis during previous episodes of febrile neutropenia.

**Figure 1:**
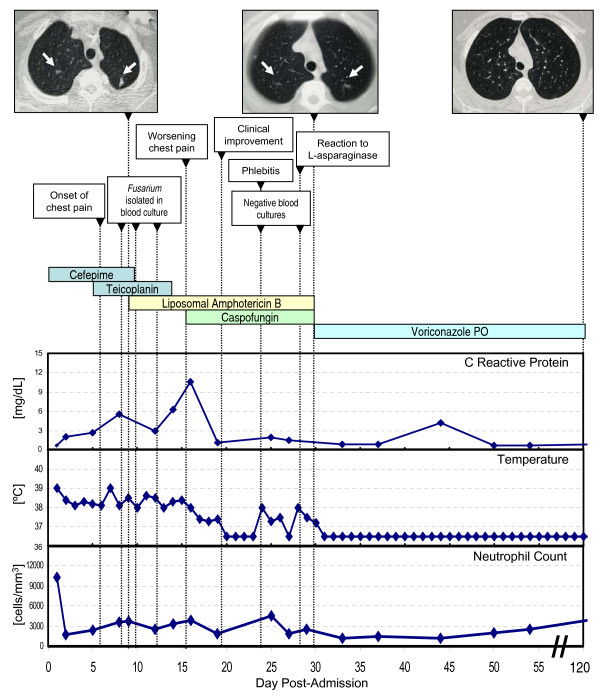
Clinical, bio logical, and radiographic evolution and drug therapy.

The physical examination was normal and the patient's general condition good. The blood examination showed 12,500 leukocytes/mm^3 ^(82% neutrophils). An urine examination, a chest X-ray, and an abdominal ultrasonography did not reveal abnormal findings. Blood, urine, and stool cultures were also negative. She was initially treated with cefepime and teicoplanin. G-CSF was discontinued, after which the neutrophil count stabilized at around 1,700/mm^3^. CRP increased steadily up to 5.6 mg/dL despite broad-spectrum antibiotic therapy. One week later, while continuously febrile, the patient experienced worsening chest pain. A chest CT scan revealed several nodules 1 cm or smaller in diameter at the apex of both lungs (Figure 1). The *Aspergillus *galactomannan antigen was negative. Cultures of separate blood samples obtained percutaneously and from the central venous catheter, yielded *Fusarium *spp. The species of the infecting *Fusarium *could not be identified and *in vitro *susceptibility could not be tested. Liposomal amphotericin B (3 mg/kg/day) was then started. Although the dose of liposomal amphotericin B was increased to 5 mg/kg daily and the central venous catheter was removed (cultures of the catheter tip were sterile), the patient remained febrile and chest pain continued to worsen over the following 7 days. Blood cultures remained positive for *Fusarium *spp. and CRP levels increased up to 10.6 mg/dL (Figure 1). Caspofungin was then initiated (70-mg load followed by 50 mg daily). Fever disappeared within 48 hours of caspofungin onset (Figure 1). Chest pain improved significantly, blood cultures became negative, and CRP levels went down to the 1–2 mg/dL range over the following days. The patient presented two isolated fever spikes 8 and 12 days after the onset of caspofungin, which corresponded to a phlebitis episode and a limited reaction to L-asparaginase, respectively. Two weeks later, a new chest CT scan demonstrated progressive resolution of the lung nodules (Figure 1). Intravenous antifungals were discontinued and chemotherapy and suppressive therapy with oral voriconazole 200 mg twice daily was started. Three months later, a chest CT scan showed complete resolution of the pulmonary lesions, while the patient was asymptomatic (Figure 1). Thus, voriconazole was discontinued. Twenty-five months after discontinuation of all antifungal therapy, the patient remains healthy in the absence of any symptoms of fungal infection and in CR of her neoplastic disease.

*Fusarium *resistance to most antifungals and the severe immunosuppression-notably long-lasting, severe neutropenia – in oncohematological patients make *Fusarium *infections commonly fatal [1]. Despite its low activity against *Fusarium *[2], amphotericin B remains the drug of choice. Voriconazole is licensed for the treatment of fusariosis based on *in vitro *data and a series of case reports with a reported response rate of ~40% [3]. Posaconazole also has potential for therapy of systemic fusariosis [4]. In any case, responses obtained with monotherapies remain too low and unsatisfactory.

We decided to treat our patient with amphotericin B and caspofungin for two reasons: (i) In our view amphotericin B remains the mainstay of therapy for fusariosis in pediatric patients; experience with voriconazole in this patient segment is limited [5-7]; (ii) Concurrent antifungal therapy is now generally considered an alternative way of improving outcome in difficult-to-treat invasive mycoses. In a recent *in vitro *study amphotericin B and voriconazole rendered mainly additive or subadditive interactions against *Fusarium *spp. [8]. Yet, the concern remains that combining amphotericin B and azoles may lead to antagonism. In addition to the azole inhibition of the synthesis of amphotericin B's pharmacological target ergosterol, amphotericin B-related damage to the fungal cell membrane may interfere with the influx of azoles [9]. *In vitro *caspofungin-inactive against *Fusarium*-showed synergistic or synergistic-to-additive interactions with amphotericin B for at least half of the *Fusarium *isolates [10]. By inhibiting cell wall synthesis, caspofungin may presumably enhance the penetration of amphotericin B [9].

We hypothesize that combination antifungal therapy likely contributed to the sucessful treatment of our patient's severe invasive fusariosis, since clinical improvement only became evident after initiating caspofungin. Yet, the effect of caspofungin in the successful outcome of our case is not completely clear. The clinical response observed could also be the result of prolonged administration of liposomal amphotericin B and the lack of severe neutropenia. Finally, the administration of suppressive voriconazole and the early re-initiation of chemotherapy allowed by rapid infection control probably also contributed to the good long-term outcome. Of note, there have been a few case reports of response to caspofungin in combination with amphotericin B [11,12] and as monotherapy [13], although in some of these reports there was a clear association of response to recovery from neutropenia.

## Conclusion

We herein report an episode of disseminated fusariosis in a pediatric ALL patient treated successfully with caspofungin and amphotericin B followed by suppressive therapy with voriconazole. While our anecdotal observation is encouraging, the use of combination antifungal therapy is debatable and further *in vivo *studies are warranted to establish its true role in deep-seated and disseminated *Fusarium *infections.

## Abbreviations

**CD10+: **Cluster differentiation 10-positive

**CT: **Computed tomography.

**SHOP-99: **The standard protocol of the Spanish Society of Pediatric Hematology and Oncology for high risk acute lymphoblastic leukemia, which includes induction (prednisone, daunomycin, vincristine, cyclophosphamide, metothrexate, L-asparaginase), consolidation (metothrexate, cytarabine, mercaptopurine), and intensification (adriamycin, vincristine, dexamethasone, cyclophosphamide, metothrexate, cytarabine, L-asparaginase) chemotherapy.

**G-CSF: **Granulocyte-colony stimulating factor.

**CRP: **C reactive protein.

## Competing interests

C. Sanz-Rodriguez is an employee of Merck Sharp & Dohme de España, S.A.. The remaining authors declare that they have no competing interests.

## Authors' contributions

J-M. Vagace, M-S. Casado, N. Alonso, M. Garcia-Dominguez, F. Garcia de la Llana, L. Zarallo, M. Fajardo, and R. Bajo participated actively in the diagnosis, care, and follow-up of the patient.

J-M. Vagace obtained consent from the patient's parents for this case report, reviewed the literature, provided clinical details, and drafted the manuscript.

C. Sanz-Rodriguez reviewed the literature and gave helpful comments regarding the scientific content of the manuscript.

All authors read and approved the final manuscript.

## Pre-publication history

The pre-publication history for this paper can be accessed here:


